# Supplementary material for: De novo Powered Air-Purifying Respirator Design and Fabrication for Pandemic Response
Source: medRxiv. 2021 Mar 29:2021.03.25.21252076. Preprint. [Version 1] doi: 10.1101/2021.03.25.21252076 (PMC8020994; doi:10.1101/2021.03.25.21252076)
Supplement: Supplement 4 — Supplementary Material 4: PanFab PAPR design materials and instructions, including: 3D-printing instructions, Arduino code, assembly instructions, bill of materials, CAD design files, custom Arduino shield design, and use instructions. [file media-4.zip › Supplementary Material 4/3D Printing Instructions/3D Printing Instructions.pdf]

3D Printing Instructions for PanFab PAPR Parts

Akshay Kothakonda[1,2,*], Lyla Atta[1,3,*], Deborah Plana[1,4,5,*], Ferrous Ward[1,2,*], Chris Davis[1,6], Avilash Cramer[1,5], Robert Moran[1,7], Jacob Freake[1,8], Enze Tian[1,9], Ofer Mazor[1,10], Pavel Gorelik[1,10], Christopher Van[1,11], Christopher Hansen[1,12], Helen Yang[1,13], Michael S. Sinha[1,13], Ju Li[1,14], Sherry H. Yu[1,15], Nicole R. LeBoeuf[1,16,†], Peter K. Sorger[1,4,†]

[1] Greater Boston Pandemic Fabrication Team (PanFab) c/o Harvard-MIT Center for Regulatory Science, Harvard Medical School, Boston, MA, USA
[2]Department of Aeronautics and Astronautics, MIT, Cambridge, MA, USA
[3]Johns Hopkins University School of Medicine, Baltimore, MD, USA
[4]Harvard Ludwig Cancer Research Center and Department of Systems Biology, Harvard Medical School, Boston, MA, USA
[5] Harvard-MIT Division of Health Sciences & Technology, Cambridge, MA, USA
[6]GenOne Technologies, Cambridge, MA, USA
[7]Mine Survival, Panama City Beach, FL, USA
[8]Fikst Product Development, Woburn, MA, USA
[9]Beijing Key Laboratory of Indoor Air Quality Evaluation and Control, Department of Building Science, Tsinghua University, Beijing, China
[10]Research Instrumentation Core Facility, Harvard Medical School, Boston, MA, USA
[11]Borobot, Middleborough, MA, USA
[12]Harvard Graduate School of Design, Cambridge, MA, USA
[13]Harvard-MIT Center for Regulatory Science, Harvard Medical School, Boston MA, USA
[14]Department of Nuclear Science and Engineering and Department of Materials Science and Engineering, MIT, Cambridge, MA, USA
[15]Department of Dermatology, Yale School of Medicine, New Haven, CT USA
[16] Department of Dermatology, Center for Cutaneous Oncology, Brigham and Women's Hospital and Dana-Farber Cancer Institute, Boston, MA, USA

[*]These authors contributed equally to this work
[†]Co-corresponding authors. E-mails: nleboeuf@bwh.harvard.edu; peter_sorger@hms.harvard.edu cc: Maureen_Bergeron@hms.harvard.edu;

ORCID IDs:
Akshay Kothakonda, 0000-0001-5424-4228
Lyla Atta, 0000-0002-6113-0082
Deborah Plana, 0000-0002-4218-1693
Avilash Cramer, 0000-0003-0014-8921
Jacob Freake, 0000-0002-5198-835X
Enze Tian, 0000-0001-6410-5360
Christopher Van, 0000-0003-3262-964X
Christopher Hansen, 0000-0002-6640-2745
Ju Li, PhD, 0000-0002-7841-8058
Helen Yang, 0000-0002-9455-5300
Michael S. Sinha 0000-0002-9165-8611

© President and Fellows of Harvard College and The Massachusetts Institute of Technology, 2020.

Sherry H. Yu: 0000-0002-1432-9128
Nicole R. LeBoeuf, MD, MPH, 0000-0002-8264-834X

Peter Sorger, PhD, 0000-0002-3364-1838

INTRODUCTION

The PanFab PAPR unit was designed in response to the personal protective equipment (PPE) shortages caused by the COVID-19 pandemic. As such, viable tested prototypes were printed in low-cost, readily available materials such as Polyethylene terephthalate – glycol (PETG) polyester polymer on a high-speed fused deposition modeling (FDM) printer. PETG was chosen as the prime material candidate based on preliminary testing for layer adhesion, total part strength, impact resistance, ease of printing, and viability of post-processing. MSLA Resin printing was used to fabricate smaller parts with tighter tolerances. The primary resin used was SirayaTech Blu resin (polyurethane – acrylate copolymer).

While all the prototypes for PanFab Custom and Commercial PAPR's used in design validation testing were 3D printed, all the parts have been optimized for injection molding for high volume production.

This document presents printing settings that were used by the PanFab team in prototyping the Custom PAPR and Commercial PAPR designs. Note that the settings and material should be optimized for the specific printer used in printing the parts.

PANFAB CUSTOM DESIGN PAPR HOUSING

The PanFab PAPR Custom Design housing has the following components:

- Lid, has integrated female NATO 40 mm threaded connecter for filters
- Bin, which attaches to it all the components to power and control the blower

<u>Bin with Gasket Recess</u>

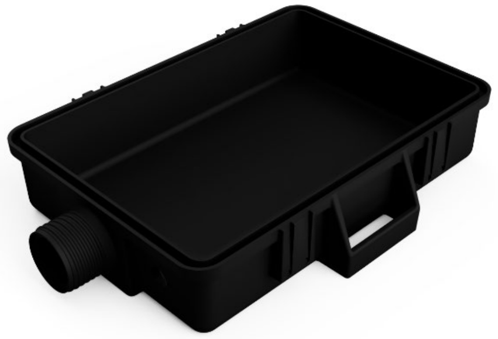

The housing parts and Milwaukee Filter Cartridge Cover were printed on a RailCore II 300ZLT FDM 3D printer. The "bin" and "lid" parts must be nominally air-tight, so all air handled by the device must first pass through filters. The printed part can maintain function even without dense infill, but top and bottom layers must be increased, and slight over extrusion (or top layer "ironing" is recommended). Layer height of 300 microns was used with 15% rectilinear infill. Increase vertical shells to 4 for increased part strength. This part is designed to minimize supports, but still requires support on the gasket seal overhang, and threaded outlet. The threads on the outlet *will* need to be manually finished, and this can be accomplished with a cylindrical file (or m3

© President and Fellows of Harvard College and The Massachusetts Institute of Technology, 2020.

threaded rod, if necessary). Additional air tightness can be provided with a conformal coating of resin or other sealant. Print in default orientation with the interior of the part facing upwards.

Lid with Tongue Feature

Settings similar to “Bottom with Gasket Recess.” Print with the surface visible in the figure to the right on the build plate (larger opening on threaded holes toward build plate)

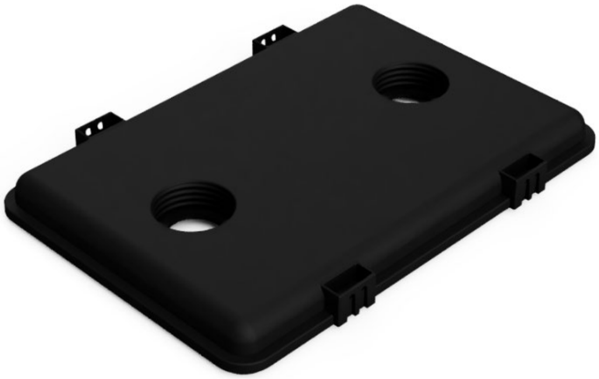

COMMERCIAL MILWAUKEE FILTER CARTRIDGE COVER

This part covers and protects the filter media of the Milwaukee commercial filter cartridge from damage. Print this part with flat surface on build plate, 100% infill, two walls. This is not a structural part, and therefore can be printed much thinner than either the bottom with gasket recess or the lid.

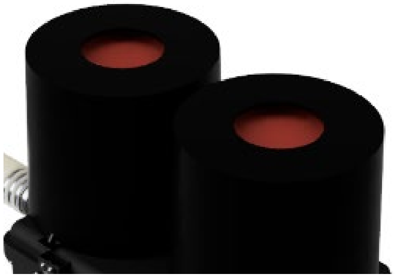

ALL OTHER PARTS

In addition to the parts discussed, there are several smaller parts such as the hose adapter, hood coupler, locking ring, filter inserts, hose insert, blower adapter, blower mounts, and belt loops. Print these in resin for optimal air tightness. Resin used was SirayaTech Blu, on Anycubic LCD 3D printers (Photon S and Mono X). The resin must be optimized for the printer being used. Parts can also be printed in FDM, but this was not tested by our team for NIOSH-equivalent tests.

© President and Fellows of Harvard College and The Massachusetts Institute of Technology, 2020.
