# Supplementary material for: De novo Powered Air-Purifying Respirator Design and Fabrication for Pandemic Response
Source: medRxiv. 2021 Mar 29:2021.03.25.21252076. Preprint. [Version 1] doi: 10.1101/2021.03.25.21252076 (PMC8020994; doi:10.1101/2021.03.25.21252076)
Supplement: Supplement 4 — Supplementary Material 4: PanFab PAPR design materials and instructions, including: 3D-printing instructions, Arduino code, assembly instructions, bill of materials, CAD design files, custom Arduino shield design, and use instructions. [file media-4.zip › Supplementary Material 4/Bill of Materials/Custom Design.pdf]

Bill of Materials for PanFab Custom PAPR Design

Akshay Kothakonda[1,2,*], Lyla Atta[1,3,*], Deborah Plana[1,4,5,*], Ferrous Ward[1,2,*], Chris Davis[1,6], Avilash Cramer[1,5], Robert Moran[1,7], Jacob Freake[1,8], Enze Tian[1,9], Ofer Mazor[1,10], Pavel Gorelik[1,10], Christopher Van[1,11], Christopher Hansen[1,12], Helen Yang[1,13], Michael S. Sinha[1,13], Ju Li[1,14], Sherry H. Yu[1,15], Nicole R. LeBoeuf[1,16,†], Peter K. Sorger[1,4,†]

[1] Greater Boston Pandemic Fabrication Team (PanFab) c/o Harvard-MIT Center for Regulatory Science, Harvard Medical School, Boston, MA, USA
[2]Department of Aeronautics and Astronautics, MIT, Cambridge, MA, USA
[3]Johns Hopkins University School of Medicine, Baltimore, MD, USA
[4]Harvard Ludwig Cancer Research Center and Department of Systems Biology, Harvard Medical School, Boston, MA, USA
[5] Harvard-MIT Division of Health Sciences & Technology, Cambridge, MA, USA
[6]GenOne Technologies, Cambridge, MA, USA
[7]Mine Survival, Panama City Beach, FL, USA
[8]Fikst Product Development, Woburn, MA, USA
[9]Beijing Key Laboratory of Indoor Air Quality Evaluation and Control, Department of Building Science, Tsinghua University, Beijing, China
[10]Research Instrumentation Core Facility, Harvard Medical School, Boston, MA, USA
[11]Borobot, Middleborough, MA, USA
[12]Harvard Graduate School of Design, Cambridge, MA, USA
[13]Harvard-MIT Center for Regulatory Science, Harvard Medical School, Boston MA, USA
[14]Department of Nuclear Science and Engineering and Department of Materials Science and Engineering, MIT, Cambridge, MA, USA
[15]Department of Dermatology, Yale School of Medicine, New Haven, CT USA
[16] Department of Dermatology, Center for Cutaneous Oncology, Brigham and Women's Hospital and Dana-Farber Cancer Institute, Boston, MA, USA

[*]These authors contributed equally to this work

[†]Co-corresponding authors. E-mails: nleboeuf@bwh.harvard.edu; peter_sorger@hms.harvard.edu cc: Maureen_Bergeron@hms.harvard.edu;

ORCID IDs:
Akshay Kothakonda, 0000-0001-5424-4228
Lyla Atta, 0000-0002-6113-0082
Deborah Plana, 0000-0002-4218-1693
Avilash Cramer, 0000-0003-0014-8921
Jacob Freake, 0000-0002-5198-835X
Enze Tian, 0000-0001-6410-5360
Christopher Van, 0000-0003-3262-964X
Christopher Hansen, 0000-0002-6640-2745
Ju Li, PhD, 0000-0002-7841-8058

© President and Fellows of Harvard College and The Massachusetts Institute of Technology, 2020.

Helen Yang, 0000-0002-9455-5300
Michael S. Sinha 0000-0002-9165-8611
Sherry H. Yu: 0000-0002-1432-9128
Nicole R. LeBoeuf, MD, MPH, 0000-0002-8264-834X

Peter Sorger, PhD, 0000-0002-3364-1838

Following are the parts used in the PanFab Custom PAPR Design. The estimated costs assume a total production run of 2000 units.

S. No.	Item	Qty.	Supplier/Method	Part No.	Estimated cost ($)
1	Housing Bin	1	ABS Injection Molding	N/A	9.60 (Tooling Cost = 10,720)
2	Housing Lid	1	ABS Injection Molding	N/A	9.70 (Tooling Cost = 19,589)
3	Blower, Centrifugal	1	Delta Electronics	BFB1012HD-04D4L	36
4	Battery pack, 12V NiMH	1	Tenergy	Amazon Standard Identification Number: B077Y9HNTF	23
5	Controller	1	Arduino	R3	23
6	PCB Shield	1	OSH Park	N/A	9.50
7	Differential Pressure Sensor	1	Sensirion	SDP810-500PA	19
8	Buzzer	1	Mallory Sonalert Products	PS-580Q	5
9	Potentiometer	1	Bourns Inc.	93R1A-R22-A12L	3
10	Transistor	1	ON Semiconductor	2N3904BU	0.20
11	Resistor	1	Vishay BC Semiconductor	PR02000201001JR500	0.32
12	Electrical connector	1	TE Connectivity AMP Connectors	1-2834184-3	1.40
13	Venturi	1	BPE Inc.	178-71-2	0.54

© President and Fellows of Harvard College and The Massachusetts Institute of Technology, 2020.

14	Venturi Ports	2	Car-Anth Manufacturing	16-1204-2	0.68 (Tooling Cost = 375)
15	Blower silicone tube	1	McMaster-Carr	3038K29	N/A
16	Venturi silicone tube	2	McMaster-Carr	3038K12	N/A
17	Blower adapter	1	ABS 3D printing	N/A	6
18	Housing Gasket	1	Apple Rubber	N/A	3.20 (Tooling Cost = 2560)
19	Housing Latches	4	McMaster-Carr	1794A55	15.20
20	Gaskets for threaded connections	4	McMaster-Carr	5647K62	3
21	Latch screws	16	McMaster-Carr	98164A441	1.55
22	Latch nuts	16	McMaster-Carr	90730A007	0.65
23	Switch	1	McMaster-Carr	8002K114	28
24	Switch Cover	1	McMaster-Carr	70205K4	4.40
25	Waist strap	1	Skil-Care	PathoShield Gait Belt	11
26	Filter	2	Custom Filters	N/A	19 (Tooling Cost = 26020)
27	Hose adapter	2	ABS Injection molding	N/A	6.60 (Tooling Cost = 9997)
28	Hose	1	Flexaust	Flex-Tube PU-IH, PN: 33800125000	8.50
29	Hood coupler	1	ABS Injection molding	N/A	3.30 (Tooling Cost = 7456)
30	Locking ring	1	ABS Injection molding	N/A	2.70 (Tooling Cost = 5156)
31	Hood	1	University of Washington	VHA ADAPT PAPR Hood	30
**Total**					**284.04**

Note: Several costs are unofficial quotes

© President and Fellows of Harvard College and The Massachusetts Institute of Technology, 2020.
