# Supplementary figures and images for: De novo Powered Air-Purifying Respirator Design and Fabrication for Pandemic Response
Source: medRxiv. 2021 Mar 29:2021.03.25.21252076. Preprint. [Version 1] doi: 10.1101/2021.03.25.21252076 (PMC8020994; doi:10.1101/2021.03.25.21252076)

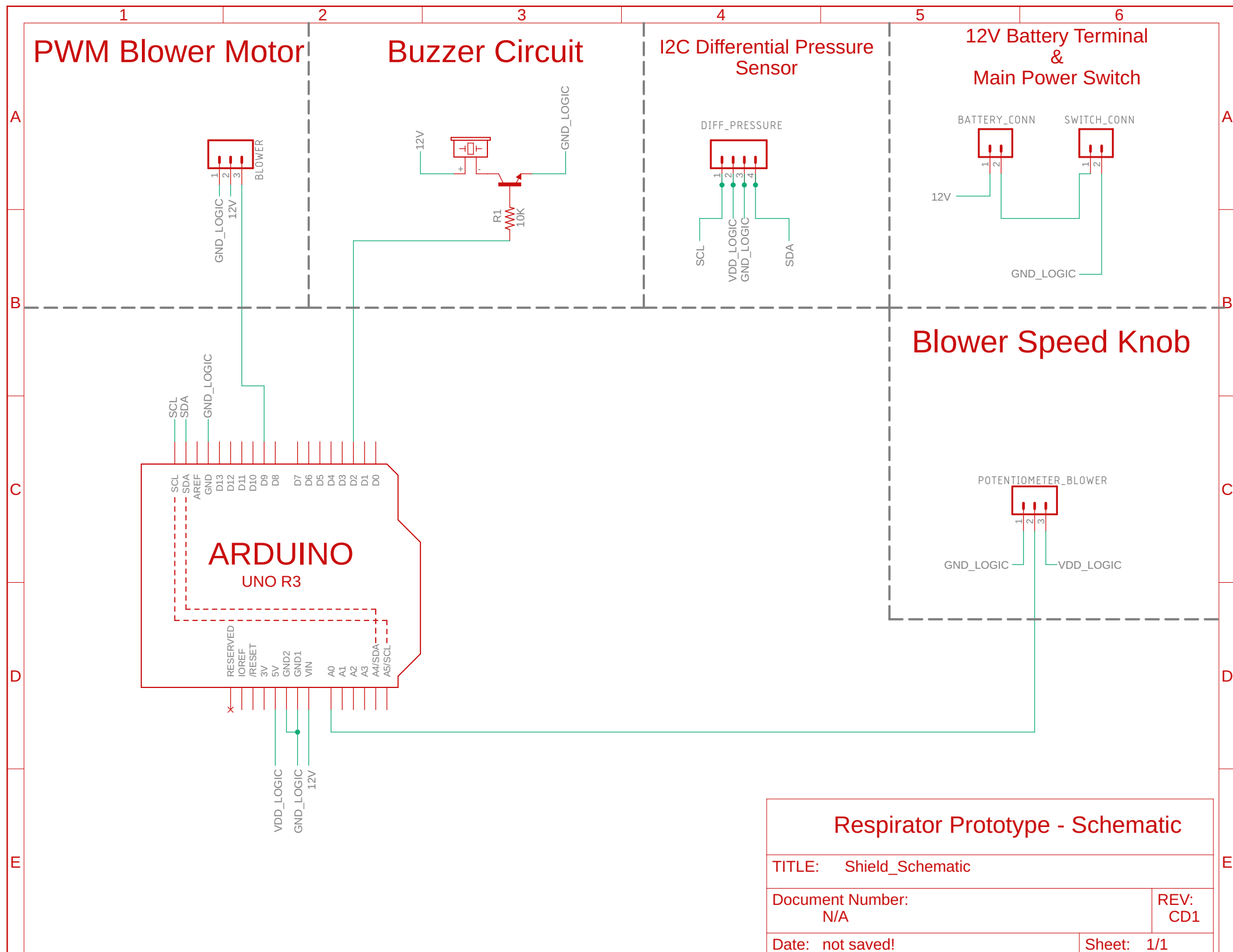

Supplement: Supplement 4 — Supplementary Material 4: PanFab PAPR design materials and instructions, including: 3D-printing instructions, Arduino code, assembly instructions, bill of materials, CAD design files, custom Arduino shield design, and use instructions. [file media-4.zip › Supplementary Material 4/Custom Arduino Shield Design/Shield_Schematic.pdf]

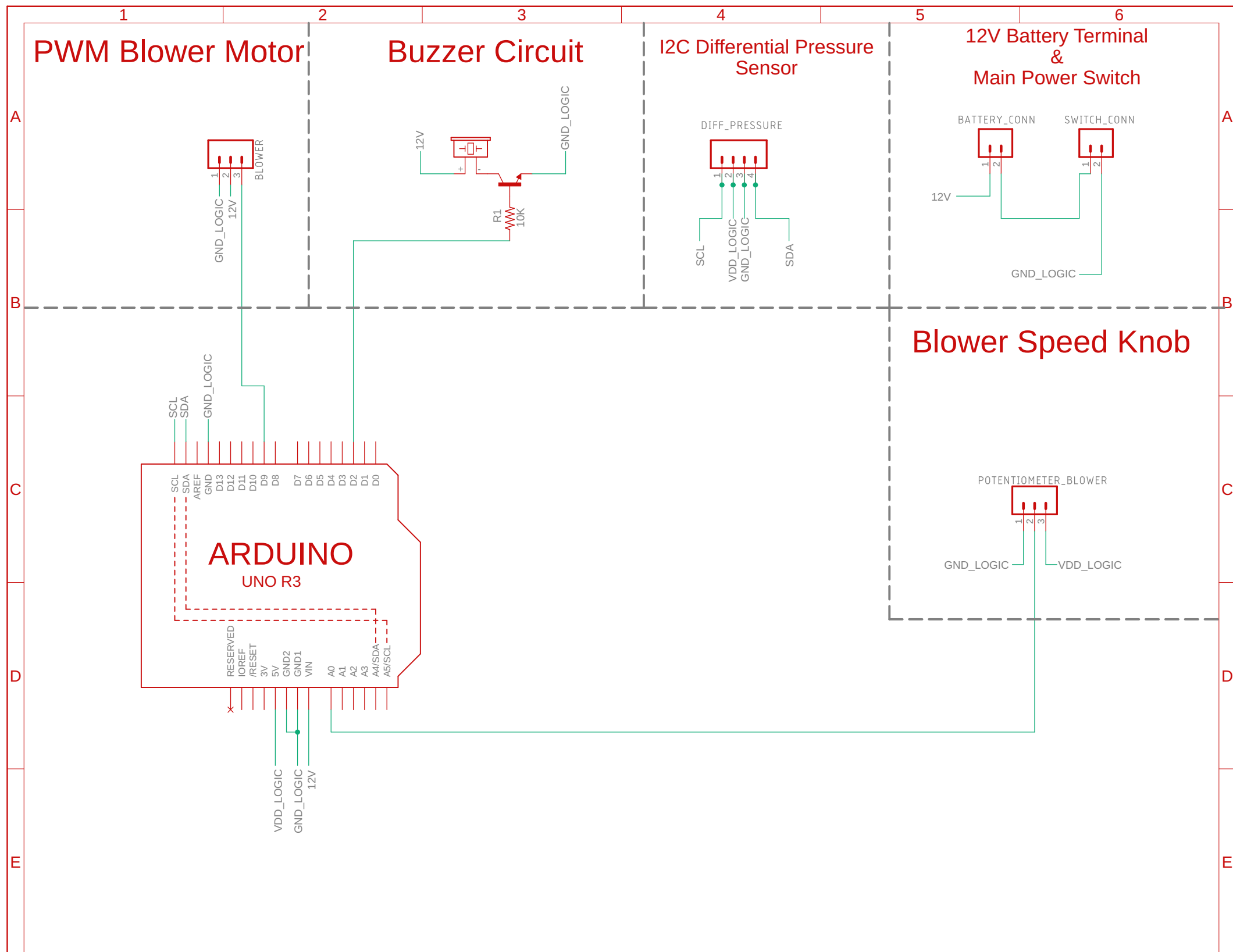

Supplement: Supplement 4 — Supplementary Material 4: PanFab PAPR design materials and instructions, including: 3D-printing instructions, Arduino code, assembly instructions, bill of materials, CAD design files, custom Arduino shield design, and use instructions. [file media-4.zip › Supplementary Material 4/Custom Arduino Shield Design/Shield_Schematic_NoInfo.pdf]
